# Diseases of the Pancreas

**Published:** 1903-08-29

**Authors:** 


### Hospital Clinics and Medical Progress.

### DISEASES OF THE PANCREAS.

In discussing the present position of our knowledge of diseases of the pancreas, Mr. Moynihan [1] draws attention to the results of experimental work on that organ. Thus it has been shown that total extirpation of the pancreas causes all the symptoms of diabetes in addition to digestive disturbances. If only part of the gland be removed diabetes does not occur. Subcutaneous grafting of a portion of the pancreas, if the graft survives, will prevent the symptoms of diabetes even if no portion of pancreas be left in the abdomen, but on removing the graft in such a case glycosuria develops.

Ligature of the duct of the pancreas does not cause the duct behind the ligature to dilate, neither does it cause diabetes, although it gives rise to chronic interstitial inflammation in the gland. The explanation of diabetic manifestations occurring under certain pathological conditions affecting the pancreas, and not under others, is that the interlobular groups of cells alone, known as the islands of Langerhaus, are those that control metabolism by means of an internal secretion, and that it is necessary for these cells to be rendered functionless before pancreatic diabetes can occur. In the pancreatitis which follows ligature of the duct these islands of Langerhans for the most part escape, and therefore glycosuria is not brought about.

The symptoms of pancreatic disease Mr. Moynihan places under six heads, namely, glycosuria, hæmorrhage, fatnecrosis, altered character of the stools, alteration in the constituents of the urine, and wasting. Taking these in order: Glycosuria, as mentioned above, is almost certainly due to alteration in the islands of Langerhaus. Hæmorrhage may occur with acute, subacute, or chronic inflammation, although any of these forms may occur without any evidence of local bleeding. Experiments have shown that hæmorrhage may be brought about by the injection into the duct of Wirsung of the bacillus diphtheriæ, or of the bacillus pyocyaneus. In certain diseases of the pancreas there may be a general hæmorrhagic tendency, which is much increased by the presence of jaundice. In the process of fat-necrosis the fat is split up into fatty acids and glycerine, the latter of which is absorbed; and one theory which has been put forward to explain the occurrence of hæmorrhage in pancreatis is that it is due to the absorption of the glycerine.

The fat-necrosis or saponification is attributed to the escape of the fat-splitting ferment of the pancreatic juice, and it has been shown in cats that by causing the whole of the secretion of the pancreas to penetrate into the surrounding tissues, saponification results, not only in the abdominal fat, but in the fat of the pericardium and subcutaneous tissue as well.

Absence of the pancreatic secretion from the intestine results in the passage of clay-coloured stools. In this connection it has been demonstrated that the colour of the fæces in health is due, not to the bile-pigments which are absorbable, but to an insoluble pigment which is formed by the action of the pancreatic juice upon some of the colouring matters of the bile. A deficiency either of the bile or of the pancreatic juice will therefore cause the fæces to be unpigmented. It is well to remember that the pancreas has two ducts, the duct of Wirsung and the duct of Santorini, and that both must be blocked, or the gland so altered that it ceases to secrete, if the above symptom is to occur. The presence of undigested muscle fibre in excess, and also of fat, is found in the stools where there is an absence or deficiency of pancreatic secretion. The main alteration to be found in the urine is a reduction of the ethereal sulphates.

With regard to the wasting, in Mr. Moynihan's experience the rapidity with which the body-weight declines in cases of pancreatic disease can only be matched in malignant disease. This is only to be expected, seeing that the pancreas is so important in the process of digestion.

After dealing with pancreatic cysts, the best treatment for which he believes as a rule to consist of evacuation and drainage, Mr. Moynihan passes on to a consideration of pancreatic calculus. The stones are generally white or of light colour, may be single or multiple, and are found in all parts of the duct, but much the most frequently in the head. The symptoms have not been thoroughly worked out up to the present. There is usually pain in the upper part of the abdomen, and it frequently resembles that due to gall-stones. When the pain is at its height, vomiting, hiccough, rigors, cold sweats, or collapse may be noticed. Diabetes has often occurred. Hauseman, in 72 cases of diabetes associated with pancreatic disturbance, found that stone was present in 12. Osler, in 70 cases of calculus, observed that diabetes was recorded 24 times. Other symptoms are marked bodily wasting and diarrhœa, while jaundice may be caused by pressure upon the common duct. Whether the stones form as a result of inflammation, or whether the pancreatitis is set up by the calculi is a debatable point.

Pancreatitis may be classified as acute, subacute, and chronic. The acute form may result in local or general hæmorrhage, in necrosis of the gland, or in suppuration. Clinically the symptoms resemble those of acute peritonitis in the epigastric region. The onset is abrupt and the progress rapid. The illness commences with sudden acute pain in the abdomen, followed by collapse and vomiting. The pulse is rapid and thin, and the temperature slightly elevated. There is no intestinal obstruction. Jaundice is often observed and is due to inflammation of the common bile duct. The illness commonly occurs in middle-aged men, and there is often a history of gall-stone trouble, or indigestion, or alcoholic indulgence.

In the most acute cases death occurs in from three to five days. If the diagnosis be made, probably the best treatment is laparotomy and drainage of the pancreatic region, preferably from behind.

Chronic pancreatitis is due to infection extending up from the intestinal canal or from the bile passages, or to pancreatic calculi or to malignant disease, either primary in the gland itself, or spreading to it from a neighbouring structure. It is also said that certain poisons circulating in the blood may set up chronic inflammation, for example, alcohol and the toxins of tubercle, and syphilis. Cholelithiasis is the most important and frequent of all these causes. Associated with gall-stone disease all stages of chronic pancreatitis are met with, but most commonly the head of the pancreas is the part most affected. In such case the head of the gland will be found thickened and harder than normal. The treatment of chronic pancreatitis consists of the performance of a cholecystostomy with prolonged drainage of the gall bladder and bile ducts.

[1] Practitioner, Aug., 1903.

